# COVID-19 fear among junior undergraduate nursing students during the pandemic in South Africa

**DOI:** 10.4102/hsag.v28i0.2371

**Published:** 2023-11-30

**Authors:** Ilze Steenkamp, Jennifer Chipps

**Affiliations:** 1 School of Nursing, Faculty of Community and Health Sciences, University of the Western Cape, Cape Town, South Africa

**Keywords:** COVID-19, pandemic, nursing students, fear, clinical practical training

## Abstract

**Background:**

During the COVID-19 pandemic, nursing students continued to work in facilities to complete clinical hours. Little was known about the impact of COVID-19 on nursing students during this time.

**Aim:**

To investigate fear of COVID-19 among junior undergraduate nursing students during the pandemic.

**Setting:**

A student nursing school at a university in the Western Cape, South Africa.

**Methods:**

A cross-sectional survey was conducted with 559 nursing students. A self-administered questionnaire with the validated COVID-19 fear scale (α= 0.84) was distributed. Scale reliability, factor analysis, means and 95% confidence intervals were calculated for items, overall scale and associations with demographic variables were tested using Kruskal–Wallis Independent Samples and Mann–Whitney U tests.

**Results:**

There were 370 respondents (68.51% response rate), predominantly female (294, 79.5%) and exhibited a mean age of 21.9 years (± 3.9). More than half, 192 respondents (51.9%) reported mild fear of COVID-19, 103 (27.8%) moderate fear and 57 (15.4%) severe fear. Apart from gender, no significant demographic associations with overall COVID-19 fear were found. Factor analysis identified two distinct factors, physiological and emotional expressions of fear (moderate significant positive correlation between factors [*r* = 0.541]).

**Conclusion:**

The study’s findings reveal that junior undergraduate nursing students, during the pandemic, generally reported experiencing mild fear related to COVID-19.

**Contribution:**

This study contributes to the field of COVID-19 fear studies, provides insight into factors influencing fear levels and validates the scale’s factor structure.

## Introduction

COVID-19 brought about a global pandemic and along with the rest of the world, South Africa imposed a national lockdown to prevent the spread of the virus. The lockdown resulted in the removal of students from campuses at universities, with the commencement of online learning. Nursing students were also removed from clinical practice during this time. However, because of the clinical learning requirements of 4000 h of practical learning in clinical facilities over the 4 years of training, after 6 months, student nurses were allowed to return in a phased manner to clinical skills laboratories on campus and clinical placement facilities.

At the time of return to clinical facilities, COVID-19 was an unknown condition, with reports of uncertainties around the impact of the pandemic and mixed information on safety requirements (Crowe et al. 2021). Factors such as the financial and social impact of COVID-19, social circumstances and taking care of one’s family can contribute to psychological stress and physiological factors such as irritability, poor sleep quality (Labrague, De Los Santos & Fronda 2022) and loneliness were linked with high levels of COVID-19 fear (Beisland et al. 2021). In preparing the students for working in healthcare facilities, an intensive orientation programme was conducted, covering information related to COVID-19, infection prevention and control, distribution of personal protective equipment (PPE) and social and emotional support (online and in-person) (Chipps et al. 2022).

It was anticipated that the returning students would experience stress and anxiety related to COVID-19 (Beisland et al. 2021; Kuru Alici & Öztürk Çopur 2022; Labrague et al. 2022; Savitsky et al. 2020). Fear is a common response to disease outbreaks and is a normal adaptive response to a perceived or actual threat (Pretorius et al. 2021), and elevated and continued physiological and emotional responses can raise levels of psychological distress and exacerbate existing mental health problems (Pretorius et al. 2021). To measure fear related to COVID-19, a brief and valid instrument, the Fear of COVID-19 Scale (FCV-19S), was developed to enable healthcare providers to plan and develop appropriate programmes to address this fear (Ahorsu et al. 2022; Lin et al. 2021).

## Aim of study

The aim of this study was to investigate fear of COVID-19 among junior university undergraduate nursing students, new to clinical practice, during the COVID-19 pandemic.

## Methods

A cross-sectional survey of undergraduate nursing (first- and second-year) students who were new to clinical practice, was conducted using a self-administered scale to investigate fear of COVID-19. The study was conducted at a nursing school at a university in the Western Cape, South Africa. The students were enrolled in a Bachelor of Nursing programme, which is required by the South African Nursing Council for registration as a registered nurse (General, Psychiatric and Community and Midwife). The survey was conducted in February and March 2021, prior to the return of first- and second-year nursing students to clinical practice to complete their clinical practical requirements. The population for this study included a total of 559 nursing students, with *n* = 252 in Year one, and *n* = 307 in Year two of the study. A minimum required representative sample of 324 (58.0%) was calculated using a margin error of 5%, confidence of 95% and response distribution at 50% and quota sampling was used to select 153 in year one (47.2%) and 171 (52.7%) in year two. The inclusion criteria for the study included nursing students who were enrolled in both the first and second year of the Bachelor of Nursing programme and there were no exclusion criteria for this group. A total of 540 questionnaires were distributed and 370 completed questionnaires were returned.

Data were collected using a self-administered questionnaire, which included demographic information, self-reported medical problems, hours of sleep and history of COVID-19 testing, and the FCV-19S (Ahorsu et al. 2022; Lin et al. 2021). The FCV-19S was developed with seven items providing self-reported levels of COVID-19 fear over the last month using a five-point Likert scale (Ahorsu et al. 2022; Lin et al. 2021). The scale ranges from 1 (strongly disagree) to 5 (strongly agree) and had established internal consistency (α = 0.84) (Ahorsu et al. 2022; Lin et al. 2021). Data were captured and analysed in SPSS v28.0 (IBM Corporation, Armonk, New York, USA). The total score was calculated by adding up each item score, with a range from 7 to 35 and higher scores indicating a greater perceived fear of COVID-19. Means and 95% confidence intervals were calculated for each item, and Kruskal–Wallis Independent Samples and Mann–Whitney U tests were used to test associations between demographic variables and FCV-19S items and FCV-19S score. Coronavirus disease 2019 fear was classified as mild, moderate or severe (7–19 = *mild fear*, 20–26 = *moderate fear* and > 27 = *severe fear)* (Faro et al. 2022). Although there are different reported classifications of mild, moderate and severe fear for the FCV-19S, a study in Brazil recommended 7–15 as *mild fear*, 16–25 as *moderate fear* and 26–35 as *severe fear* (Metwally Elsayed & Ahmed Ghazi 2021). The COVID-19 fear classification of Faro et al. (2022) was chosen to represent this study’s COVID-19 fear categories on account of the large sample of respondents participating (*n* = 1000) in their study (Faro et al. 2022).

To assess the reliability of the FCV-19S, Cronbach’s *α* coefficients were calculated and 0.70 or higher was adopted as the cut-off. The seven items of the FCV-19S were subject to principal component analysis (PCA) using SPSS v28.0 (Pallant 2020). The specific steps as described by Pallant (2020) included: (1) extracting data by PCA; (2) testing whether the data collected in this study were suitable for factor analysis by using the Kaiser–Meyer–Olkin (KMO) test (> 0.6), Bartlett’s test of sphericity (< 0.05) and correlation coefficients (> 0.3); (3) determine whether the data reflected the contents of all components by determining the number of factors with eigenvalues (> 1), scree plot, and the cumulative percentage of variance; (4) rotating the component matrix using varimax with Kaiser normalisation to find the best analytical result and, when the rotation converged after a certain number of iterations, acquiring the symptom-related factors and their factor loadings; and (5) analysing common factors by their clinical character. Prior to performing PCA, the suitability for factor analysis was assessed. Inspection of the correlation matrix revealed the presence of many coefficient > 0.30. The KMO test value was 0.840 and Bartlett’s test of sphericity output *p* < 0.001, supporting the factorability of the correlation matrix. Hence, PCA and factor analysis are valuable for further analysis.

### Ethical considerations

The Humanities and Social Science Research Ethics Committee (HSSREC) of the University of the Western Cape approved the methodology and ethics of the research (reference no.: HS20/10/17). All procedures performed in the study involving human participants were in accordance with the ethical standards of the institutional and/or national research committee and with the 1964 Helsinki Declaration and its later amendments or comparable ethical standards. Written informed consent was obtained from all participants involved in the study.

In an effort to ensure the well-being of all the students that participated in this study, the researchers proactively notified the students about the availability of support resources. This notification served as a reminder of the existing supporting structures and resources, encouraging students to seek assistance for COVID-19 fear-related concerns, while maintaining their anonymity.

## Results

### Demographics

A total of 540 questionnaires were distributed and 370 completed questionnaires were returned (68.51% response rate), with a mean age of 21.9 years (± 3.9, median age = 21, range 18–43 years). Most of the respondents were female, 294 (79.5%), with 160 (43.2%) first year and 204 (55.1%) second-year respondents. Very few of the respondents lived on their own (28, 7.6%) with over half of the respondents reporting living with family, friends, or roommates (188, 50.8%), and 145 living in university residences (39.2%) (Table 1). Thirty-three (8.9%) respondents indicated that they had a known chronic illness and the average hours of sleep of the respondents were 7.12 h (± 1.4) (range 3–12 h) per night. Of the respondents, 28 (7.6%) had taken a COVID-19 test with 10 receiving a positive COVID-19 test and 14 (3.8%) reporting that they had reached out to support structures during the COVID-19 pandemic (Table 1).

**TABLE 1:** Demographics and COVID-19 fear risk factors.

Demographic factors	Mean	SD	Range	*n*	%
**Age (362)**	21.88	±3.89	18–43	-	-
**Gender (365)**					
Male	-	-	-	71	19.2
Female	-	-	-	294	79.5
**Year of study (364)**					
First Year	-	-	-	160	43.2
Second Year	-	-	-	204	55.1
**Living arrangements (361)**					
Alone	-	-	-	28	7.6
With others	-	-	-	188	50.8
University residences	-	-	-	145	39.2
**COVID-19 fear risk factors (370)**					
COVID-19 Testing	-	-	-	28	7.6
COVID-19 Diagnosis	-	-	-	10	2.7
**Chronic diseases (369)**	-	-	-	33	8.9
**Seeking support (369)**	-	-	-	14	3.8
**Hours of sleep (365)**	7.12	±1.43	3–12	-	-

### Fear of COVID-19 Scale validity and factor structure

**FIGURE 1:**
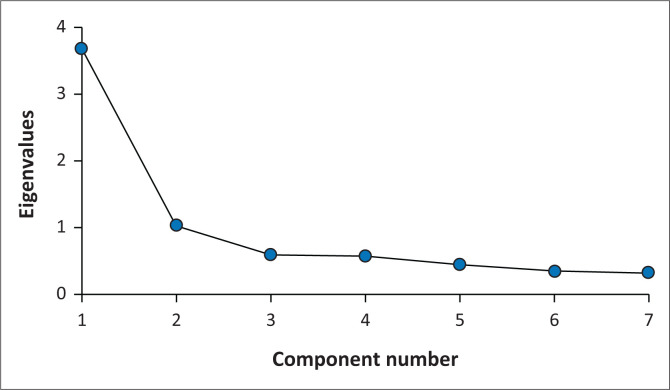
Scree plot of components and their eigenvalues.

The FCV-19S reliability was confirmed in this study with a Cronbach’s alpha of α = 0.85. Principal component analysis yielded two factors with eigenvalues greater than 1 (Table 2), confirmed by scree plot (Figure 1). The cumulative percentage of variance was 67.3%, which shows that these factors could well represent the whole data. The Promax rotation, which was performed three times to obtain the factor loading matrix, revealed the two factors with both factors showing strong loadings and all variables also loading on one factor, except for item 7 (each factor included several variables with a loading factor greater than 0.3) (Table 2). There was a moderate positive correlation between the two factors (*r* = 0.541). Through factor analysis, these two common factors were classified as Affective and Physiological responses.

**TABLE 2:** Means and component matrix for principal component analysis (two factors extracted) (*n* = 354).

Fear of COVID-19 scale items	Mean	SD	Item-total correlation	Factors		Communality
				Affective	Physiological	
I am most afraid of COVID-19	3.45	1.36	0.792	0.848	-	0.642
It makes me uncomfortable to think about COVID-19	3.05	1.39	0.775	0.595	-	0.568
My hands become clammy when I think about COVID-19	2.01	1.25	0.773	-	0.758	0.669
I am afraid of losing my life because of COVID-19	3.70	1.49	0.722	0.889	-	0.693
When watching news and stories about COVID-19 on social media, I become nervous or anxious	3.06	1.41	0.721	0.455	0.430	0.604
I cannot sleep because I’m worrying about getting COVID-19	1.77	1.15	0.649	-	0.988	0.799
My heart races or palpitates when I think about getting COVID-19	2.14	1.36	0.634	-	0.833	0.737
**Total FCV-19S**	**19.2**	**6.81**	**-**	**-**	**-**	**-**

*Source:* Ahorsu, D.K., Lin, C.Y., Imani, V., Saffari, M., Griffiths, M.D. & Pakpour, A.H., 2022, ‘The fear of COVID-19 scale: Development and initial validation’, *International Journal of Mental Health Addiction* 20, 1537–1545. https://doi.org/10.1007/s11469-020-00270-8; and Lin, C.Y., Hou, W.L., Mamun, M.A., Aparecido Da Silva, J., Broche-Pérez, Y., Ullah, I. et al., 2021, ‘Fear of COVID-19 Scale (FCV-19S) across countries: Measurement invariance issues’, *Nursing Open* 8(4), 1892–1908. https://doi.org/10.1002/nop2.855

FCV-19S, Fear of COVID-19 Scale.

### COVID-19 *fear*

The mean total FCV-19S score was 19.2 (± 6.81)/35 with just over half of the respondents categorising as having mild fear (*n* = 192, 51.9%), followed by moderate fear (*n* = 103, 27.8%) and severe fear (*n* = 57, 15.4%). There was no significant difference in the respondents’ overall mean score of fear based on demographic variables, COVID-19 testing and chronic illnesses (*p* > 0.05).

### Affective factors

**FIGURE 2:**
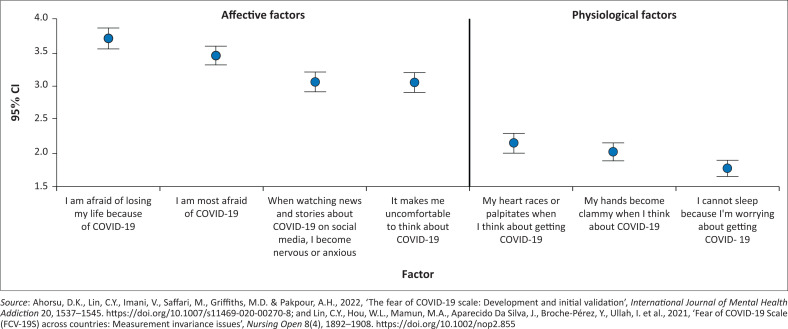
Individual factors in the fear of COVID-19 Scale (95% confidence intervals).

Using the two factors identified in the factor analysis, affective factors included four emotional statements of fear (Figure 2 and Table 3). ‘I am afraid of losing my life because of COVID-19’ (3.70 ± 1.49) was rated significantly higher than all other items, except for ‘I am most afraid of COVID-19’ (3.45 ± 1.35). This was followed by ‘When watching news and stories about COVID-19 on social media, I become nervous or anxious’ (3.06, ± 1.41), and lastly ‘It makes me uncomfortable to think about COVID-19’ (3.05 ± 1.38), which was rated significantly higher by respondents over the age of 21 years (3.29 ± 1.36 vs. 2.84 ± 1.38, *K* = 8.02, *p* = 0.018). Respondents with < 7 h of sleep per night rated ‘I am most afraid of COVID-19’ lower than respondents with > 7 h of sleep per night (3.48 ± 1.55 vs. 3.81 ± 1.43, *K* = 8.87, *p* = 0.012). Lastly, male respondents rated ‘When watching news and stories about COVID-19 on social media, I become nervous or anxious’, significantly lower than females (2.69 ± 1.27 vs. 3.15 ± 1.42, *U* = 2.44, *p* = 0.015).

**TABLE 3:** Fear of COVID-19 Scale item mean score (*n* = 354).

FCV-19S items	Mean	SD
I am most afraid of COVID-19	3.45	1.36
It makes me uncomfortable to think about COVID-19	3.05	1.39
My hands become clammy when I think about COVID-19	2.01	1.25
I am afraid of losing my life because of COVID-19	3.70	1.49
When watching news and stories about COVID-19 on social media, I become nervous or anxious	3.06	1.41
I cannot sleep because I’m worrying about getting COVID-19	1.77	1.15
My heart races or palpitates when I think about getting COVID-19	2.14	1.36
**Total FCV-19S**	**19.2**	**6.81**

*Source*: Ahorsu, D.K., Lin, C.Y., Imani, V., Saffari, M., Griffiths, M.D. & Pakpour, A.H., 2022, ‘The fear of COVID-19 scale: Development and initial validation’, *International Journal of Mental Health Addiction* 20, 1537–1545. https://doi.org/10.1007/s11469-020-00270-8; and Lin, C.Y., Hou, W.L., Mamun, M.A., Aparecido Da Silva, J., Broche-Pérez, Y., Ullah, I. et al., 2021, ‘Fear of COVID-19 Scale (FCV-19S) across countries: Measurement invariance issues’, *Nursing Open* 8(4), 1892–1908. https://doi.org/10.1002/nop2.855

FCV-19S, Fear of COVID-19 Scale.

### Physiological factors

Physiological factors included three statements of fear expressed physiologically, which were rated significantly lower than the affective factors (Figure 2 and Table 3). The highest rated physiological item was ‘My heart races or palpitates when I think about getting COVID-19’ (2.14 ± 1.36), which was significantly higher than the lowest rated item ‘I cannot sleep because I’m worrying about getting COVID-19’ (1.77, ± 1.15) (Figure 2 and Table 3). This item was rated significantly higher by respondents > 21 years (1.90 ± 1.18 vs. 1.61 ± 1.09, *K* = 9.04, *p* = 0.011). This was also confirmed by year level with respondents in the year two, similarly rating this item significantly higher (1.88 ± 1.19 vs. 1.60 ± 1.06, *U* = 2.68, *p* = 0.007). Similarly, rating the item ‘My heart races or palpitates when I think about getting COVID-19’, respondents in the second year of the nursing programme rated this higher, although not significant (2.28 ± 1.41 vs. 1.95 ± 1.26, *U* = 2.23, *p* = 0.26).

## Discussion

### Factor structure

The PCA found support for a two-factor model, namely affective, and physiological response, which were moderately correlated (*r* = 0.541). This supports similar findings where two factors and a strong one factor model were identified (Balazs, Mitev & Brodszky 2022, Masuyama, Shinkawa & Kubo 2022). The internal consistency and reliability for the FCV-19S in this study was found to be high (α = 0.85), which was similar to studies performed in other countries and South Africa where COVID-19 fear was investigated among nursing students using the FCV-19S such as Egypt, α = 0.82, Turkey, α = 0.91 (Kuru Alici & Ozturk Copur 2022), Thailand α = 0.84 (Karawekpanyawong et al. 2022) and South Africa α = 0.88 (Chipps et al. 2022).

### COVID-19 *fear*

In this study, the total score of the FCV-19S was 19.20 (± 6.81) out of a possible 35, indicating mild fear of COVID-19 during the pandemic. These findings were similar to other nursing student studies such as: Turkey (19.35 ± 5.90) (Nehir & Gungor Tavsanli 2021), Egypt among first year nursing students (17.96 ± 5.10) (Metwally Elsayed & Ahmed Ghazi 2021) and South Africa among fourth year students (20.27 ± 7) (Chipps et al. 2022). The total FCV-19S score reported in this study may be because of the orientation programme offered by the university that included support from academic and clinical staff together with information of COVID-19, which reduced their feelings of anxiety (Chipps, Jarvis & Brysiewicz 2021, Jarvis et al. 2021). First and second year students also returned to the platform after the third- and fourth-year students, which may have reassured them.

Affective factors rated through the items ‘I am afraid of losing my life because of COVID-19’ (3.70 ± 1.49) and ‘I am most afraid of COVID-19’ (3.45 ± 1.35) were rated significantly higher than the physiological factors. This finding is similar to other studies where the item ‘I am most afraid of COVID-19’ was rated much higher than the other items on the FCV-19S (Chi et al., 2022; Faro et al. 2022; Winter et al. 2020). It is also hypothesised that fear of COVID-19 can be because of the recollection of past disease outbreaks (Van Damme & Van Lerberghe 2000). The association between fear of COVID-19 expressed through affective symptoms, supported the important impact of COVID-19 on psychological and mental health (Han, Park & Lee 2021).

Female respondents rated fear higher with a mean score of the FCV-19S of 19.48 (± 6.87) compared with male respondents (17.73 ± 6.14). Numerous other studies also confirmed higher ratings on the FVC-19S in female respondents (Ding et al. 2021; Maslakçı & Sürücü 2022; Metin et al. 2022), which may be because of previous findings that women are more psychologically vulnerable than men to the COVID-19 pandemic (Ding et al. 2021). Studies conducted among university students indicated that female respondents experienced greater levels of anxiety and even more so in students who are in the field of health sciences such as nursing (Alhadi & Alhuwaydi 2021; Martínez-Lorca et al. 2020). Coherently with a study performed among the general population in China, females exhibited higher anxiety associated with the psychological impact of the pandemic (Wang et al. 2020).

A study investigating the use of mobile technology in South Africa during the COVID-19 lockdown period reported that 89.6% of the participants in the study stated that the lockdown had forced them to use more mobile technology, with social media usage being the second highest (Fischer et al. 2021). There is evidence that information on social media may amplify panic among people (Shehata & Abdeldaim 2022), as this is a widely used platform for information access (Shehata & Abdeldaim 2022). In comparing gender ratings on the item ‘When watching news and stories about COVID-19 on social media, I become nervous or anxious’, males rated this item significantly lower than females (2.69 ± 1.27 vs. 3.15 ± 1.42, *U* = 2.44, *p* = 0.015). This finding could be because of the various reports of higher occurrences of anxiety and stress disorders in women according to a study performed in the US investigating depression, anxiety and stress regarding COVID-19 fear (Maslakçı & Sürücü 2022). Heffner, Vives and Feldmanhall (2021) found that females in the general population of the US that have high levels of anxiety consume a considerable amount of COVID-19-related media information from social media platforms such as Facebook and Twitter. The findings of Heffner et al. (2021) are congruent with findings in Ghana reporting that large social media usage among female respondents increases COVID-19 fear levels (Malm et al. 2022). Various studies reported that the excessive usage of social media during the COVID-19 pandemic was linked to high levels of anxiety among university students (Alhadi & Alhuwaydi 2021; Jiang 2021; Mengistu et al. 2023). Mobile phone usage and accessing social media applications such as Facebook and Twitter drastically increased during the pandemic; access to a mobile device was important for students using online platforms for learning and social interactions (Jiang 2021; Mengistu et al. 2023).

There were no other significant associations among the total FCV-19S score and demographic factors, which was similar to other findings reporting that age groups, classes, place of residence and employment status did not affect the fear of COVID-19 (Mamuk & Akarsu 2022). However, when comparing the ratings of the item ‘It makes me uncomfortable to think about COVID-19’ by different age groups, it was rated significantly higher by respondents over the age of 21 years. Studies have reported that thoughts of COVID-19 drive uneasiness in respondents and contribute to an increased level of COVID-19 fear among respondents who are in their 20s (Sit et al. 2021; Son et al. 2020). In this study, over half of the respondents indicated that they live with others; thus, higher COVID-19 fear scores for some items on the FCV-19S could be related to some sort of family responsibility they have at home towards others; similar findings were reported in Kuru Alici & Öztürk Çopur (2022); Laranjeira et al. (2021); Roldán-Merino et al. (2022). The restrictions imposed by the pandemic together with prolonged anxiety caused by the fear of COVID-19 can lead to depression among nursing students (Laranjeira et al. 2021; Roldán-Merino et al. 2022; Sakai et al. 2022).

The lowest scoring item in FCV-19S in this study was, ‘I cannot sleep because I’m worrying about getting COVID-19’ (1.77 ± 1.15) and similar results were found in other studies (Chi et al. 2022; Chia, Oyeniran & Iorfa 2021; Faro et al. 2022). In this study respondents indicated an average of 7.12 (± 1.4) h of sleep per night, which is the recommended hours of sleep in the adult population (Hirshkowitz et al. 2015). However, respondents who reported less than 7 h of sleep per night rated the item, ‘I am most afraid of COVID–19’, significantly lower compared with those who reported more than 7 h of sleep per night (3.48 ± 1.55 vs. 3.81 ± 1.43, *K* = 8.87, *p* = 0.012). Studies have found that a high proportion of respondents experienced abnormal sleeping patterns such as increased hours of sleep and poor sleep quality related to the COVID-19 pandemic (Güven & Altay 2022; Son et al. 2020).

The results of this study were cross-sectional and provide an insight into COVID-19 fear and the relation thereof to psychological well-being among junior university students studying nursing. Results from this study show that fear of COVID-19 is positively associated with psychosocial distress. The study was completed almost 2 years into the pandemic, which could have an effect on the COVID-19 fear and psychological well-being; at the time of the study, the vaccine roll out was in the implementation phase and junior students were involved in an extensive training and information programme prior to going back into the healthcare facilities. These factors might have increased or decreased COVID-19 fear and psychological well-being.

### Limitations

The target population of the study was limited to first- and second-year students. The data were collected from only one faculty of nursing in the Western Cape, South Africa; therefore, the results may not be generalised to all nursing students in South Africa.

### Recommendations

There will be future pandemics and preparedness for students is crucial. Further studies are needed to determine the effect of the pandemic with a larger sample of students and the effects on students beyond the pandemic. A larger sample will allow more understanding in how students dealt with the pandemic and their coping mechanisms. With future studies the aim could be towards the development of policies and protocols to help and support students and lessen the anxiety of clinical placement during outbreaks, epidemics and pandemics.

## Conclusion

The study investigated the fear of COVID-19 among nursing students using the FCV-19S that was developed by Ahorsu et al. (2022) and Lin et al. (2021). In this study, most of the respondents fell into the mild fear of COVID-19 category. The authors found no statistically significant differences in the overall mean score of the FCV-19S in terms of demographic variables; however, when individual items of the FCV-19S were compared, the authors found statistically significant differences when the same demographic variables were compared. Policies and protocols that are dedicated to support students during and after a life-changing occurrence, such as the COVID-19 pandemic, should be in place and function without difficulty.
